# A record review of reported musculoskeletal pain in an Ontario long term care facility

**DOI:** 10.1186/1471-2318-6-5

**Published:** 2006-03-23

**Authors:** Connie J D'Astolfo, B Kim Humphreys

**Affiliations:** 1 Department of Graduate Education and Research, Canadian Memorial Chiropractic College, Toronto, Ontario Canada

## Abstract

**Background:**

Musculoskeletal (MSK) pain is one of the leading causes of chronic health problems in people over 65 years of age. Studies suggest that a high prevalence of older adults suffer from MSK pain (65% to 80%) and back pain (36% to 40%). The objectives of this study were:

1. To investigate the period prevalence of MSK pain and associated subgroups in residents of a long-term care (LTC) facility.

2. To describe clinical features associated with back pain in this population.

3. To identify associations between variables such as age, gender, cognitive status, ambulatory status, analgesic use, osteoporosis and osteoarthritis with back pain in a long-term care facility.

**Methods:**

A retrospective chart review was conducted using a purposive sampling approach of residents' clinical charts from a LTC home in Toronto, Canada. All medical records for LTC residents from January 2003 until March 2005 were eligible for review. However, facility admissions of less than 6 months were excluded from the study to allow for an adequate time period for patient medical assessments and pain reporting/charting to have been completed. Clinical data was abstracted on a standardized form. Variables were chosen based on the literature and their suggested association with back pain and analyzed via multivariate logistic regression.

**Results:**

140 (56%) charts were selected and reviewed. Sixty-nine percent of the selected residents were female with an average age of 83.7 years (51–101). Residents in the sample had a period pain prevalence of 64% (n = 89) with a 40% prevalence (n = 55) of MSK pain. Of those with a charted report of pain, 6% (n = 5) had head pain, 2% (n = 2) neck pain, 21% (n = 19) back pain, 33% (n = 29) extremity pain and 38% (n = 34) had non-descriptive/unidentified pain complaint. A multivariate logistic regression analysis revealed that osteoporosis was the only significant association with back pain from the variables studied (P = 0.001).

**Conclusion:**

Residents with back pain represent 13.6% (n = 19) of the sample population studied. This is as frequent as other serious conditions commonly found in LTC. Of the variables studied, only osteoporosis and the self-report of back pain were found to be associated. The back pain resident in this facility can typically be described as female, osteoporotic, with mild to moderate dementia, an independent or assisted walker having low levels of depression. Further research using other sites is needed to determine the overall prevalence of this condition and its impact on quality of life issues. The results of this study should inform future research in this area.

## Background

Musculoskeletal (MSK) pain is a significant burden on the Canadian health care system. It is considered the third most expensive disorder in terms of expended health care dollars, surpassed only by cancer and heart disease [1]. The most common MSK complaint in the elderly population is back pain, second to joint arthritis [2-4]. In Ontario, back pain is the 3^rd ^leading cause of chronic health problems in the over 65 year old category for women and the fourth leading cause of chronic health problems for men in the same age group [5]. The estimated cost of back pain in Ontario is 2.4 billion dollars per year and its prevalence is estimated at 64% of the adult population per year [1,6-11]. The prevalence of pain in the elderly is not accurately known, some studies suggest that older adults have an even higher prevalence of MSK pain, between 65%–85% [12,13] with 36% to 70% reportedly suffer from a back pain condition [2-4,13].

Older adults aged 65 plus, are the fastest growing segment of the Canadian population. By 2011, the number of people age 65 and older is expected to rise to over 1.9 million [13]. By 2050 it is projected that the ratio of people 65 years or older to those 15–64 years of age, worldwide, will double and that 1 in 5 people will be in the age 65 plus category [2]. With this increasing age comes an associated increase in chronic daily pain [2,12,14]. A U.S. national survey of patients aged 75 and older demonstrated that back pain is the third most frequently reported symptom and may well be the reason for physician visits. [15] In another study, 17.3% of total back problem visits occurred in the 65 years and older age group [15,16]. Edmond et al conducted a study on 1037 surviving members (aged 68–100) of the Framingham Heart Study and found that 22.3% of elders experience back pain everyday; low back pain being the most prevalent. They also found that elders confined mostly to their homes had an especially high prevalence of back symptoms [3].

Although there is variability, the literature reports that 45–80% of long term care (LTC) geriatric residents have substantial MSK pain that is under-reported and inadequately managed [2,4,12,13,17-25]. The literature also suggests several variables that may be well associated with MSK pain in the aging population such as age, gender, a medical history of osteoarthritis, depression, osteoporosis, and difficulty ambulating (i.e., wheel chair bound or requiring assistance) [21,26-34].

Detection and management of MSK conditions is a growing health care concern as our population continues to age and as healthcare costs surmount. It is vital that MSK pain conditions, including back pain, are identified as soon as possible and non- pharmaceutical strategies implemented as an integral part of care plans for the geriatric long-term care (LTC) patient in the management of MSK pain [2,24,25,35,36]. The first study in the U.S. to determine the quality of chronic pain care provided to older persons was conducted recently by Chodosh et al. The authors evaluated quality indicators for chronic pain in a random sample of 372 older community dwelling patients using medical record review and interviews. They concluded that chronic pain management in older vulnerable patients is inadequate and that improvement is needed in screening, clinical evaluation, follow up and attention to potential toxicities of therapy. [37]. There is empirical evidence of the associations between the prevalence of MSK pain and physical as well as psychological disability in the older adult. Consequences of poorly managed pain in this population may include depression, social isolation, sleep disturbance, decreased ambulation and increased healthcare utilization and costs [2,37]. As a management strategy, the literature supports the integration of a conservative MSK pain specialist, within a collaborative pain management team [35]. Currently, however, the integration of a spinal care specialist in LTC homes is in its infancy with little research conducted in this area.

The primary objectives of this study were to:

1. To investigate the period prevalence of MSK pain and associated subgroups in residents of a LTC home.

2. To describe clinical features associated with back pain in this population.

3. To identify associations between variables such as age, gender, ambulatory status, analgesic use, cognitive status, osteoporosis and osteoarthritis with back pain in a LTC home.

The results of this study may inform future research in this area.

## Methods

A retrospective chart review of residents from a 250-bed LTC home in Toronto, Ontario was conducted. This study was approved by the home's ethics review board. Residents of various ages and medical status are integrated throughout the facility, thus every wing has essentially similarly distributed patient types as it relates to medical and functional status. It was the intent of the investigators to try to include in the study as many of the medical records as possible. However, it was necessary to limit the inclusion criteria to only records that were 6 months or older. Consequently, a purposive sampling approach was utilized. This selection process was chosen in an effort to allow for an adequate period of time for the necessary patient medical assessments and pain reporting/charting to occur as well as address resident facility turnover at that time. Although this would result in a smaller sample size for this type of analysis, resulting in reduced statistical power, the authors felt that excluding recent records (less than 6 months) enabled a more reliable picture of the long term care resident at this home.

All medical records from each of the four wings were therefore initially reviewed and charts indicating an admission date less than 6 months (110 charts) were excluded from the study, resulting in a non randomized sample size of 140 (purposive sampling). Charting methods in LTC typically include scheduled nursing and medical assessments and volunteered complaints either to physician, nurses or healthcare aids found throughout the medical file. Thus, all sections of the chart were reviewed for data including progress notes (which includes nursing and medical assessments), problem sheets (medical diagnoses), nursing daily records, physician's orders, resident quarterly assessments, medical administration records, interdisciplinary team assessments and conference records and outcome evaluations. Twenty-minutes were allocated for each chart review. Data concerning age, gender, length of stay, report of pain, pain location, analgesic use, depression, cognitive status, ambulatory status and co-morbidities were abstracted on a standardized form. An examination called the Mini Mental Status Exam (MMSE) is routinely used to assess cognitive function at this facility. The results of the MMSE were used to classify the degree of cognitive impairment among the LTC residents. The MMSE is based on a scoring range from 0–30 with lower scores indicating greater mental impairment. The following categories and ranges were used: 0–10 (severely impaired); 11–19 (moderately impaired); 20–25 (mild dementia); 26–30 (cognitive/coherent).

Variables were chosen based on the literature and their suggested correlation with MSK and back pain. The numbers of co-morbidities for each case were also included in the data abstraction and were identified from attending physicians' diagnoses, ICD 9 codes, and diagnostic assessments and test results. The charts were selected by one reviewer (CD) and examined from the period of January 2003 to March 2005. For quality assurance (accuracy of the review/abstraction process), 15 charts were randomly selected and independently reviewed for a second time and then compared to the original data extracted.

Prior to the study, the investigators predicted a lower period prevalence of residents would have a report of MSK or back pain in this study compared to that reported in the literature. Reasons for a hypothesized lower occurrence include the smaller sample size due to the exclusion of recent admissions and the method of case ascertainment. This study attempted to identity whether or not the resident had MSK pain and their subgroups on the basis of whether it was recorded in their medical record. In order to be recorded in the medical record, residents would have complained to a healthcare aid, nurse or a doctor that they have a pain complaint or in some way demonstrated pain behaviour (for those who were cognitively challenged) to a care giver/provider. Consequently it was hypothesized that this would lead to a lower occurrence of pain than if one had individually assessed and examined the LTC resident and charted the presence and diagnosis/aetiology of the pain. Nevertheless, this study sample provides a realistic description of the prevalence of MSK and back pain as it is currently being reported and charted at this home.

The objective of the study was to identity residents with recorded MSK pain and its associated subgroups. MSK pain was defined as pain originating from the MSK system, specifically mechanical in origin and not originating from visceral or cardiovascular disease, rheumatic disease or malignancies. Subgroups of MSK pain (including head pain, neck pain, back pain and extremity pain) were identified specifically from the location and description of the pain complaint recorded and through exclusion of any possible co-morbid malignancy or visceral disorder as a cause of the pain. It was therefore assumed through process of exclusion, that the pain most probably had a MSK origin. Any pain complaint which was unidentifiable as purely mechanical in origin through the medical record was grouped in a separate category, nonspecific pain. Those with no pain reported in their charts throughout the period were classified as having no pain.

All statistical analyses were performed with SPSS version 12.0 for windows. Distribution and summary statistics were examined for all variables and inconsistency checks were also performed. Descriptive statistics were used to summarize the residents' characteristics between study subgroups. These categorical variables were then analyzed via multivariate stepwise regression to determine any significantly associated variables for the report of back pain, no pain or extremity pain.

## Results

There were no inconsistencies identified from the quality review process and no missing data for the cohort. Of the 140 residents, 20% of patients were admitted between 6 months to 1 year, 28% had been living at the home for more than one year, 40% between 2 and 6 years and 12% had been residents for grater than 6 years. The mean age of the entire home was 83.3; the mean age of the study cohort was 83.7; the mean age of the back pain group was equivalent to the study group. Gender demographics and the number of co-morbidities were also similar across all groups. (Table 1)

**Table 1:** A comparison of clinical and demographic data for all study groups

	**LTC population n = 250, as recorded at admission**	**Study Sample n = 140 residents**	**No Pain Complaint Group n = 50**	Non Specific Pain Complaint Group *** n = 35	Extremity Pain Group**** n = 30	**Back Pain Group n = 19**	**Neck and Head Pain Group n = 6**
**Mean Age:**	83.25	83.7	83.8	84.5	83.2	83.7	80.5
**Minimum**	47	51	51	69	65	70	53
**Maximum**	104	101	98	101	95	98	94
**Gender:**							
**Female**	62%	69.3%	60%	75%	75%	72%	83%
**Male**	38%	30.7%	40%	25%	25%	28%	17%
**Cognitive Status:**							
**Coherent**	Unavailable*	3.6%	0%	0%	0%	7%	16.6%
**Mild Dementia**	Unavailable*	26.4%	32%	22.2%	35.7%	12%	16.6%
**Moderate Dementia**	10%	43.6%	40%	36.1%	42.9%	70%	50%
**Severe Dementia (Alzheimer's)**	2%	26.4%	28%	38.9%	14.3%	11%	16.6%
**Ambulatory Status:**							
**Independent**	38%	8.6%	12%	5.6%	3.6%	12%	16.6%
**Requires Assistance**	39%	31.4%	34%	22.2%	32.1%	47%	16.6%
**Wheel chair**	23%	59.3%	54%	69.4%	64.3%	40%	66%
**Bedridden**	0%	0.7%	0%	2.8%	0%	0%	0%
**Analgesic Use**	39%	57%	8%	77.8%	89.3%	82%	100%
**Diagnosis of Depression**	13%	16.4%	14%	25%	17.9%	5.9%	60%
**Diagnosis of OA**	9%	25%	6%	19.4%	57.1%	35.3%	50%
**Diagnosis of Osteoporosis**	17%	27.9%	20%	30.6%	25%	64.7%** (p = 0.001)	0%
**# of co-morbidities: Mean ± SD**	4.5 ± 2	5 ± 3	4.5 ± 2	5 ± 2	5 ± 3	4.6 ± 2	4 ± 2

*Total LTC population data were abstracted from initial admitting diagnosis and reflects resident's diagnostic information prior to LTC admission. Data on levels of cognitive impairment were inconsistent and unavailable.

**Multivariate regression analysis. Significant association at the p = 0.001 level

*** Reported pain complaint which is nonspecific or undetermined (i.e. MSK (mechanical), malignancy or visceral)

**** Extremity Pain includes shoulder/arm and knee pain

### Period prevalence: general pain and back pain

The results from this study identified a general pain period prevalence of 64% (n = 89) with a 40% (n = 55) prevalence of MSK pain. Of those with a pain report charted, 6% (n = 5) had head pain, 2% (n = 2) neck pain, 21% (n = 19) back pain, 33% (n = 29) extremity pain and 38% (n = 34) had non-descriptive/un-identified pain complaint. In all cases where the admitting complaint was pain, the problem remained unresolved.

A stepwise multivariate logistic regression analysis was performed between the subgroup with back pain, those without pain, those with extremity pain and head/neck to control for known confounders. The only variable that appeared to be significantly associated was back pain and osteoporosis (p = 0.001). (See Table 1) Although there are no other significant differences between the groups (i.e., age, gender, co-morbidities, ambulatory and cognitive status), some findings are worth noting. In particular, 8% of the "pain free" group was taking prescribed analgesics and 20% of those who do not report pain were diagnosed with osteoporosis (a variable that appears to be highly associated with back pain). Sixty percent of the extremity pain group has a diagnosis of osteoarthritis and 80% were in the mild to moderate level of dementia. Of those with nonspecific/unidentified pain, 22% were not prescribed analgesics for their pain, whereas 100% of those with back pain and extremity pain were prescribed analgesics. Seventy percent of those residents with unspecified pain were wheelchair bound and 40% had severe cognitive dysfunctions. (See Table 1.)

## Discussion

This retrospective chart review study provides a general description of back pain sufferers and its period prevalence within a sample of residents within a LTC home. As a comparison, a review of the facility diagnosis database revealed 8% of total residents were diagnosed with diabetes, 6% have atherosclerotic heart disease, 36% have hypertension and 13% were diagnosed with depression. Although the above conditions are typically viewed by clinicians as more life threatening and are given clinical priority, the occurrence of back pain (13.6%) appears to be just as common. These results suggest the need for better charting, recording and identification of patient pain complaints, as back pain is likely under-reported.

The study sample for chart review (56%) was similar to the total resident population in the facility in terms of demographics, i.e. age (83.7 years versus 83.3 years) and gender (69.3% female versus 62% female). However, there were notable differences between the total resident population and the study cohort with relation to severe dementia (2% versus 26.4%) and ambulatory status (38% independent walkers versus 8.6% independent walkers) respectively. However, since the total resident population data were taken from initial admission documents and the study population reflects a group of these residents, months to years post-admission, this discrepancy may be explained at least in part by the deterioration in health status associated with advancing age in long term care residents [39,40].

Due to the small sample size, the results have limited generalizability but could serve as a starting point for future work. The results of this study indicate that residents with back pain compromise 13.6% of the study sample and 21% of those who reported pain. For those with back pain, 72% are female and 11% suffer with severe dementia (Alzheimer's disease) in comparison to 62% female and 26.4% having severe dementia in the entire study sample. In addition, 35.3% of back pain residents have a diagnosis of osteoarthritis and 64.7% have osteoporosis. Although all patients in the back pain subgroup had reported and charted complaints of back pain, 84% were prescribed analgesics. Analgesics were typically prescribed as a 'prn' (take as needed) by the attending physician. Given the higher percentage of back pain sufferers that have or develop moderate dementia, 'prn' may not be the most effective way to prescribe medication for these patients. Additionally, it appears that only 11% of the back pain subgroup was independent walkers while 50% needed assistance for ambulation (38% are in wheelchairs). In future, it may be interesting to look at possible associations between back pain and immobility.

Although there appears to be a significant correlation between back pain and clinical depression in the literature [30,41], only 5.9% of the patients studied were diagnosed with depression. However, this sub group appears to have a considerable number of co-morbidities (mean of 4.6 ± 2 medical conditions) in addition to their back complaint, including some of the most prevalent disorders (osteoporosis, hypertension, dementia, and diabetes). It is of interest to note that 50% of the entire LTC cohort was found to have between 3 to 4 co-morbidities and 30% had 5 to 10 medical conditions. An interesting focus of future work may be to identify the prevalence of back pain and whether it increases with increasing number of co-morbidities.

### Limitations to this study

This retrospective record review study, due to necessary chart exclusions, resulted in a small sample size; reducing statistical power. Data abstraction from the patient record, as with all administrative data, also has inherent limitations. For example, not all information is gathered in the same way and is often limited in description. Information documented is sometimes inconsistent, untimely and incomplete. Much of the diagnoses from the patient record are based on self reporting and there are no validity studies of either clinical or self report for location of pain in the elderly patient [13]. Cognitive problems (comprehension, memory and pain recall) may also influence the reporting of back pain [39]. Depression may lead to either a decrease or increase of patient pain reporting [30,41]. In addition decreased pain perception or increased pain tolerance (stoicism) can affect back pain prevalence rates. Language and cultural barriers, proxy reporting, illiteracy, inactivity (avoidance of pain provoking activities), attitudes towards pain (stoicism), patient physical/sensory impairments, complexity and co-morbidity of existing health conditions (respondent focus on other more life threatening health issues), lack of standardized terminology, diagnostic procedures and skilled clinicians for MSK pain conditions are also complicating factors [13,17-20,30,35,42,43].

Notwithstanding the above difficulties in retrieving valid data from chart reviews in an institutionalized geriatric population, this study provides hitherto unreported information to inform future studies on back pain in this population.

## Conclusion

Back pain appears to be as prevalent as other serious conditions commonly found in long term care. Of the variables studies, only osteoporosis and the self-report of back pain were found to be associated. The back pain resident in this facility can be typically described as female, osteoporotic, with mild to moderate dementia, an independent or assisted walker and with low levels of depression. Although there are problems in retrieving accurate information from chart reviews, under-reporting of pain in general and back pain specifically is likely in this population. Further research including multiple sites is needed to determine the overall prevalence of this condition and its impact on quality of life issues.

## Competing interests

The author(s) declare that they have no competing interests.

## Authors' contributions

CJD conceived the study, participated in the study design, did the literature search, performed the data collection and statistical analysis and drafted the manuscript. BKH participated in the design of the study, consulted on the statistical analysis and interpretation of data, participated in the drafting and critical revision of the manuscript. All authors read and approved the final manuscript.

## Pre-publication history

The pre-publication history for this paper can be accessed here:


